# Standoff pump-probe photothermal detection of hazardous chemicals

**DOI:** 10.1038/s41598-020-71937-4

**Published:** 2020-09-14

**Authors:** Ramesh C. Sharma, Subodh Kumar, Abhishek Parmar, Mohit Mann, Satya Prakash, Surya N. Thakur

**Affiliations:** 1 Defence Research and Development Establishment, DRDO, Gwalior, 474002 India; 2 Laser Science and Technology Centre, Metcalfe House, Delhi, 110054 India; 3 Banaras Hindu University, Varanasi, 221005 India

**Keywords:** Optics and photonics, Applied optics, Lasers, LEDs and light sources, Optical techniques

## Abstract

A novel pump-probe Photothermal methodology using Quartz Tuning Fork (QTF) detector has been demonstrated for the first time. A tunable mid-IR Quantum Cascade Laser (QCL) and a CW fixed wavelength visible laser have been used as the pump and probe beam respectively. The developed Photothermal (PT) technique is based on Quartz Tuning Fork (QTF) detector for the detection of hazardous/explosive molecules adsorbed on plastic surface and also in aerosols form. PT spectra of various trace molecules in the fingerprinting mid- infrared spectral band 7–9 µm from distance of 25 m have been recorded. The PT spectra of explosives RDX, TNT and Acetone have been recorded at very low quantities. Acetone is the precursor of explosive Tri-Acetone Tri-Phosphate (TATP). The experimentations using pump and probe lasers, exhibit detection sensitivity of less than 5 μg/cm^2^ for RDX, TNT powders and of ~ 200 nl quantity for Nitrobenzene (NB) and Acetone (in liquid form) adsorbed on surfaces, from a distance of ~ 25 m. The sensitivity of the same order achieved from a distance of 15 m by using only a mid-IR tunable pump laser coupled to QTF detector. Thus the pump-probe PT technique is more sensitive in comparison to single tunable QCL pump beam technique and it is better suited for standoff detection of hazardous chemicals for homeland security as well as for forensic applications.

## Introduction

The detection of life threatening/harmful chemicals, such as explosives, nerve agents and other toxic materials is of paramount interest for safeguarding lives as well as important establishments. In the present scenario, the release of these chemicals cannot be ruled out as the terror threats are looming large. The impact of such incidents can be neutralized/minimized only with the timely detection from safe standoff distances. The two most popular standoff sensing techniques are Infrared spectroscopy and Raman spectroscopy. But there are several issues associated with these techniques which are the major impediments to the development of these in to reliable systems especially for defence and security applications^1^. Other popular technique, laser induced breakdown spectroscopy (LIBS) is limited in the detection range^2^. The terahertz spectroscopy is an emerging candidate for detection of explosives and hazardous chemicals but unavailability of high power compact room temperature terahertz sources makes it difficult to convert the technique into practical standoff detection system^3^. Quartz-enhanced laser photoacoustic spectroscopy (QE-LPAS) technology is fast evolving as a potential tool for standoff detection of terror threats^4–9^. The photoacoustic methodology, in general, using mid-IR wavelength and microphone detector has been discussed by many researchers^10–16^. Zrimsek et al. have demonstrated standoff photoacoustic spectroscopy of trace explosives from a meter distance using a sensitive microphone and 213 nm laser^17^. In their research, they found that the formation of gaseous species due to the photochemistry of explosives enhances the photoacoustic signal strength but at the same time reduces the lifetime of the signal in case of detection in trace quantity of analytes. As we will see in the following sections, the QE-LPAS methodology seems to be more sensitive since the Q value of the detector is very high (~ 10,000) and also have the potential to be converted into a practical system^4,6^. The Q value can be enhanced further by placing it inside a low pressure cell.

The present work aims at devising a pump-probe PT technique with the same detector that is used, in the QE-LPAS single beam technique i.e. QTF. However, the standoff detection of traces of hazardous molecules, with the PT technique, is possible from longer distances and with enhanced sensitivity. In present technique, the pump laser does not require to cover the round trip (source to sample and back to the detector). Only the visible laser has to make the round trip. Thus the availability of visible lasers with higher powers makes the system capable to detect from longer ranges. To the best of our knowledge this PT method has been developed for the first time where both the pump and probe beams are coupled to a single quartz tuning fork (QTF) detector. Further, the point/short distance standoff detection of hazardous molecules, with the PT technique, has also been carried out for comparison with the standoff PT detection.

## Experimental

**Figure 1:**
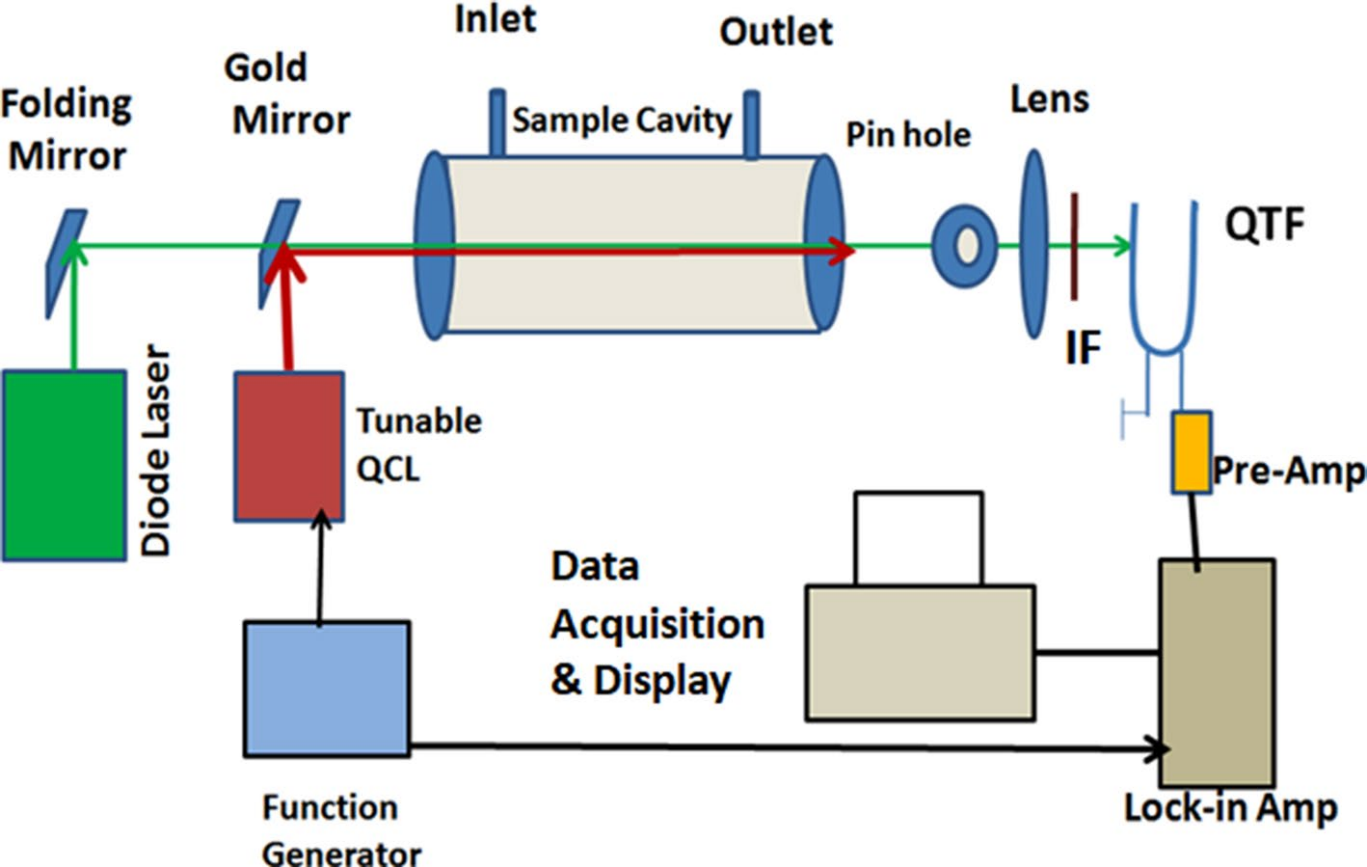
Experimental setup with vapour cell photothermal point detection of gases and aerosols with Pump-probe lasers.

**Figure 2:**
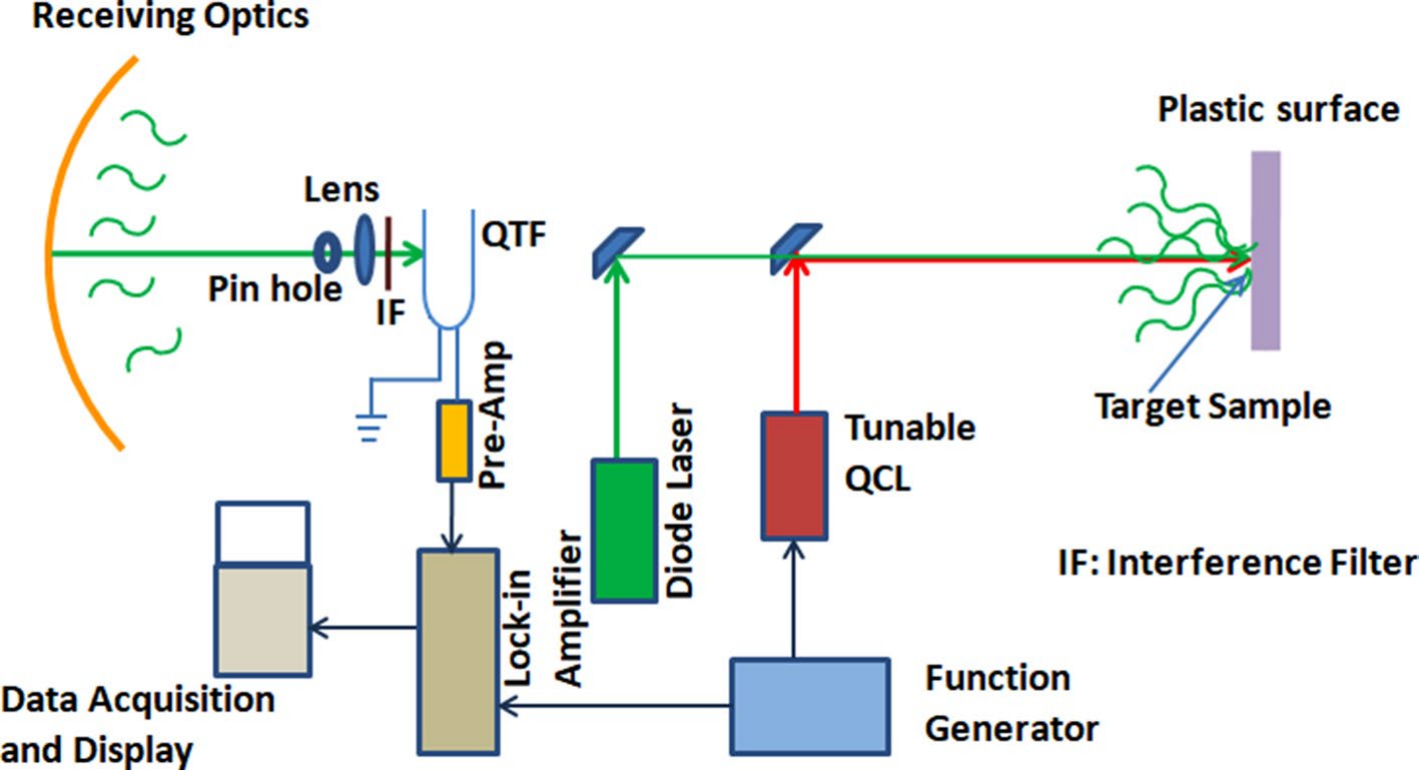
Experimental setup for standoff photothermal detection with the Pump-probe lasers.

The schematic of pump-probe PT point-detection (sample in aerosol form) is shown in Fig. 1. A pulsed tunable quantum cascade laser (QCL) pump beam was used to excite target molecules and a CW probe beam was used to observe the density fluctuation, resulting from temperature variations (due to absorption of pulsed QCL pump beam). The experimental setup for standoff detection using the PT technique is shown in Fig. 2.

The QCL beam was modulated at ~ 32.8 kHz (same as the resonant frequency of QTF detector) and both the pump and probe laser beams were co-aligned, with the latter focussed on to the QTF detector (see Figs. 1, 2). The commercially available QCL (make: M/s. Daylight Solutions; model number: Uber Tuner UT-8) having a tuning range of 7–9 μm in the mid-IR and the peak power of ~ 650 mW was used in the experiments. The QCL emits pulses of 500 ns duration at the repetition frequency of ~ 32.8 kHz using a function generator and a reference signal at the same frequency is fed into the lock-in-amplifier (Figs. 1, 2). The absorption of tunable QCL beam, incident on the target molecules of explosive species and hazardous chemicals, is followed by their non-radiative relaxation leading to density fluctuations at the modulation frequency. A 532 nm visible diode pumped solid state CW laser (Lasever model-LSR532ML, 300 mW) was used as the probe laser. The co-aligned visible probe laser-beam gets deviated from its path while passing through this region of density fluctuation, created by pump QCL, and its intensity at the pin-hole varies with wavelength of the pump beam at the modulation frequency (~ 32.8 kHz). The intensity variation of probe beam focused onto the QTF detector generates the PT signal as a function of wavelength via the piezoelectric effect. In the case of standoff detection the scattered probe (visible) beam was collected by a reflective telescope of diameter ~ 12 cm (Fig. 2). The telescope mirror is spherical enhanced aluminium coated mirror having focal ratio of (f/1.66) and FOV of 2.5 mR. The detector was placed at the focal point of the telescope. The focused beam is allowed to pass through the pin hole and focused on to the QTF. A glass lens is used to focus the probe beam tightly on the detector. The focal length of the lens used was 20 mm and the clear aperture was nearly 10 mm.

The amplitude of oscillation of the QTF prong (or tine) is proportional to the intensity of the probe laser hitting its surface and this in turn generates the PT signal to be amplified by a 10 Mega ohm pre-amplifier. An interference filter was used to transmit the 532 nm probe beam to the QTF but it blocked the pump beam from hitting the detector. The PT signal is processed by a transducer for current amplification and the amplified signal is fed into a lock-in-amplifier. The analog signal from lock-in amplifier card is digitized using a multifunction data acquisition card (USB 4716 Advantech) to be recorded on a computer with a Lab view based graphical user interface.

Measured quantities of hazardous chemicals were dissolved in a known volume of acetone to prepare the sample to be deposited as a uniform dried layer on the target surface. Chemical samples measuring 0.5 to 2.0 mg were dissolved in 10 ml of acetone and spread over the target surface area of 100 cm^2^. After vaporization of acetone from the target surface, the residue forms a thin layer of the chemical (TNT or RDX) to be analyzed by the PT sensor. The concentration was estimated by dividing the total quantity of the samples dissolved in the Acetone by the area over which it was spread (considering the residue on the surface to be nearly uniform). Solutions were prepared so as to deposit chemicals in the range of 5–20 μg/cm^2^ on targets under investigation. The target material in the present case was plastic surface. Standoff photothermal spectra of Acetone in aerosol form and liquid were also recorded in at a distance of 25 m. In case of aerosol, the aerosol cell shown in Fig. 1 was used. The cylindrical aerosol cell was 15 cm in length and 6 cm in diameter. The cell was kept along the laser beam and a plastic surface was used to cover the farther face of the cell. The laser was allowed to enter from the open end of the cell. In case of liquid Acetone the measured quantity of Acetone was poured in the thin plastic sachet. The sachet was held vertically on the axis of coaligned laser beam with the help of a plastic plate and adhesive tape.

## Results and discussion

**Figure 3:**
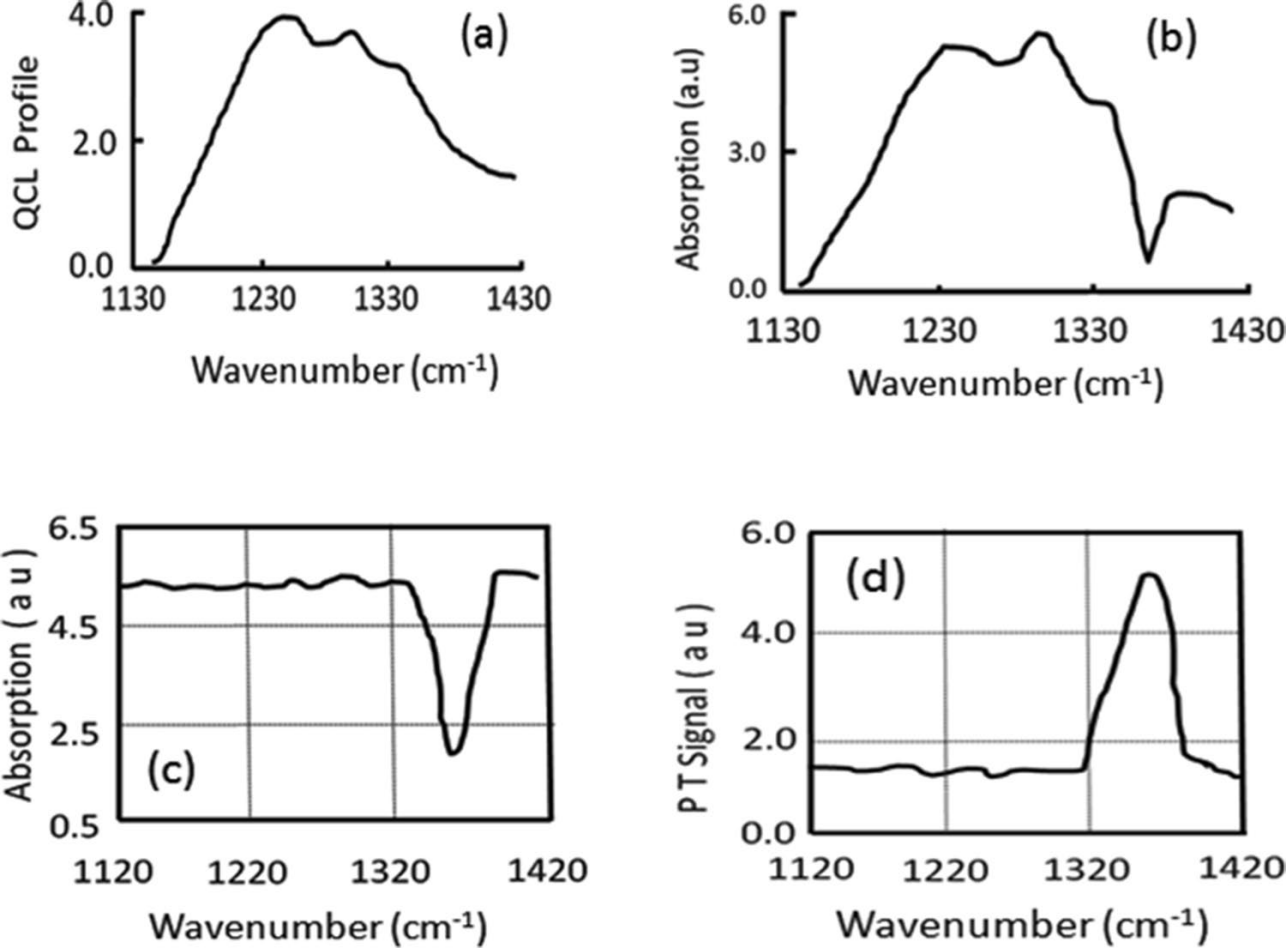
(**a**) QCL spectral profile, (**b**) Absorption spectrum of nitrobenzene, (**c**) Normalized absorption of nitrobenzene, and (**d**) Photothermal spectrum of nitrobenzene.

Tunable QCL emission profile between 7 and 9 μm (1,430 cm^−1^ to 1,130 cm^−1^), shown in Fig. 3a, has been used for generating the PT signals following absorption by molecular species on the target. Nitrobenzene has been used to illustrate the principle of PT detection with QTF. The raw absorption of nitrobenzene (NB) molecule is recorded in Fig. 3b using the QTF detector and the normalized absorption spectrum shown in Fig. 3c. The normalization is done by dividing the spectrum of nitrobenzene by spectrum of laser profile. The normalization takes care of absorption by water molecules present in the atmosphere. The observed NB absorption peak between 1,360 and 1,370 cm^−1^ is in agreement with its reported value in the literature corresponding to symmetric NO_2_ vibration of C-NO_2_ moiety in the molecule^18^.

**Figure 4:**
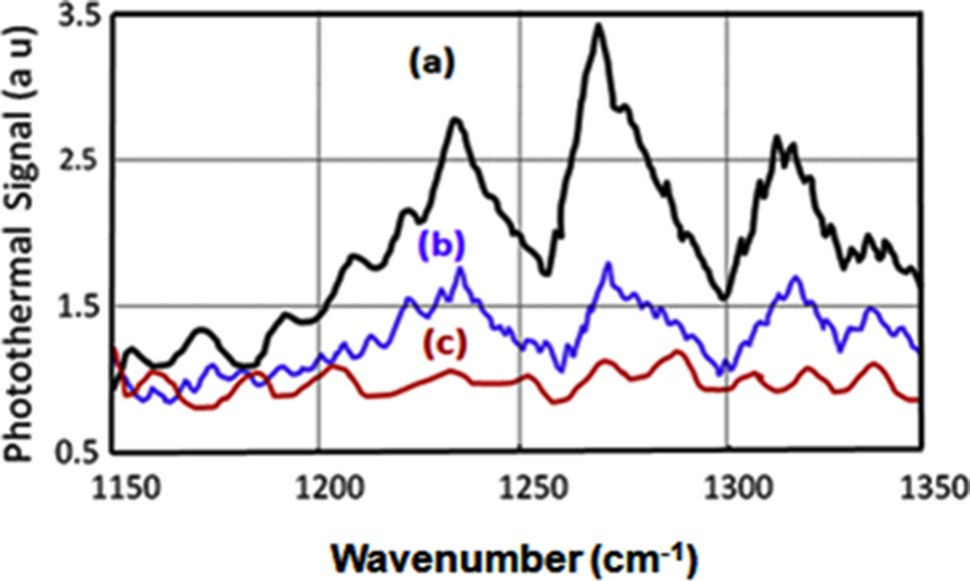
QTF photothermal signal from RDX samples by (a) Point PT detection, (b) Standoff PT detection (sample concentration: 10 µg/cm^2^), and (c) Background PT signal without sample.

**Figure 5:**
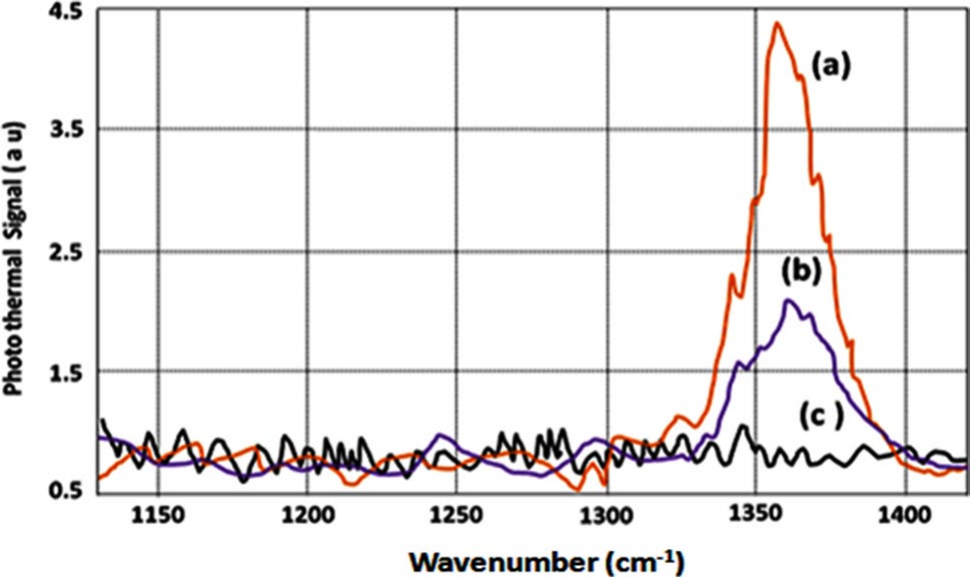
QTF photothermal signal from TNT samples by (a) Point PT detection, (b) Standoff PT detection (sample concentration: 10 µg/cm^2^) and (c) Background signal without sample.

**Figure 6:**
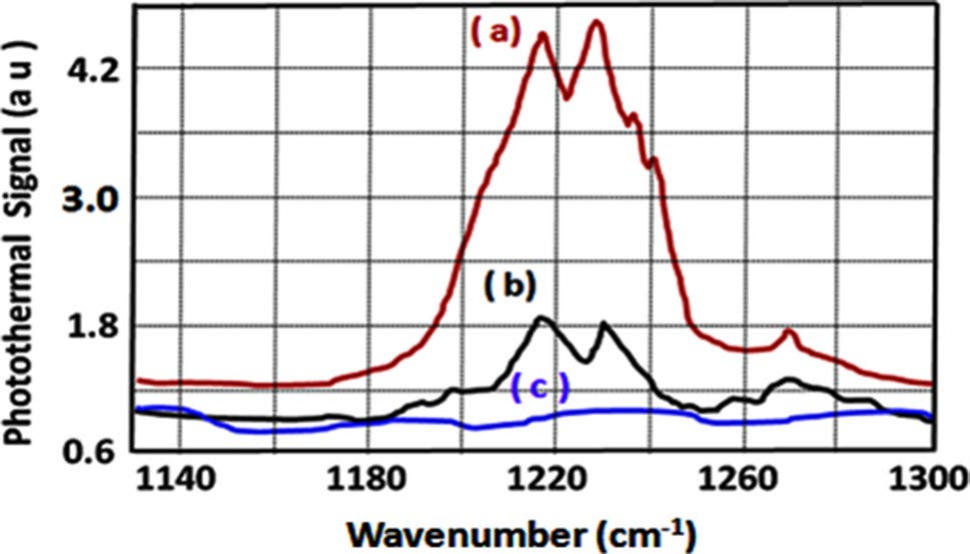
QTF photothermal signal from actone—the TATP precursor by (a) Point PT detection, (b) Standoff PT detection (sample quantity: 400 nl) and (c) Background signal without sample.

Using the experimental setup of Fig. 1 in point detection, the PT spectrum of NB molecule is plotted in Fig. 3d. The short standoff (~ 1.0 m) PT spectra of RDX, TNT and Acetone were recorded using the setup shown in Fig. 2. In these experiments, slight adjustment of the distance between telescope mirror and the detector had to be made along the axis as the focal spot slightly shifts away from the mirror as we take the target closer to the telescope. The spectra thus recorded from the short distance are shown in Figs. 4a, 5a, and 6a respectively.

The experimental setup of Fig. 2 has been used to record the standoff PT spectra of RDX, TNT, and Acetone from 25 m. These spectra are shown in Figs. 4b, 5b, and 6b respectively. Figures 4c, 5c, and 6c respectively depict the spectra recorded when there was no sample present at the target surface (or inside the aerosol cell). The scattered probe laser radiation from the target is collected by a spherical mirror of 12 cm diameter, coupled to a pinhole as shown in Fig. 2. The probe laser beam, incident on the pinhole, is focused by a lens on the QTF detector to generate the PT signal as a function of wavelength when QCL is scanned over its spectral profile. As described earlier the QCL radiation is blocked, from hitting the QTF, by the 532 nm interference filter. The strongest peaks are resolved at 1,268 cm^−1^ for RDX molecule, and at 1,364 cm^−1^ of TNT molecule in agreement with the literature^19^.

Acetone is the precursor of TATP explosive and its PT spectra, shown in Fig. 6, exhibit two strong peaks at 1,218 cm^−1^ and at 1,230 cm^−1^ in agreement with the normal modes of vibration corresponding to ‘CCC bend + OCO bend’ and ‘CC stretch + CCO bend’ respectively^20^.

It is apparent from the vibrational assignments described above^18–20^, that the molecular species are clearly detected by the PT sensor. The strength of noise signal, corresponding to the baseline (spectrum from the clean surface, without sample) of Figs. 4, 5 and 6, was about 75 mV while a value of 150 mV for the PT signal was taken as the detection limit and this gives a SNR of about 2. Noise level was taken as the average height of the spectra of clean surfaces (without sample). For calculating noise level, those spectral ranges were used where the noise were maximum. The detection limit is found to be 200 nl concentration for Acetone and 5 μg/cm^2^ for TNT and RDX with a SNR ~ 2 at a distance of 25 m.

**Figure 7:**
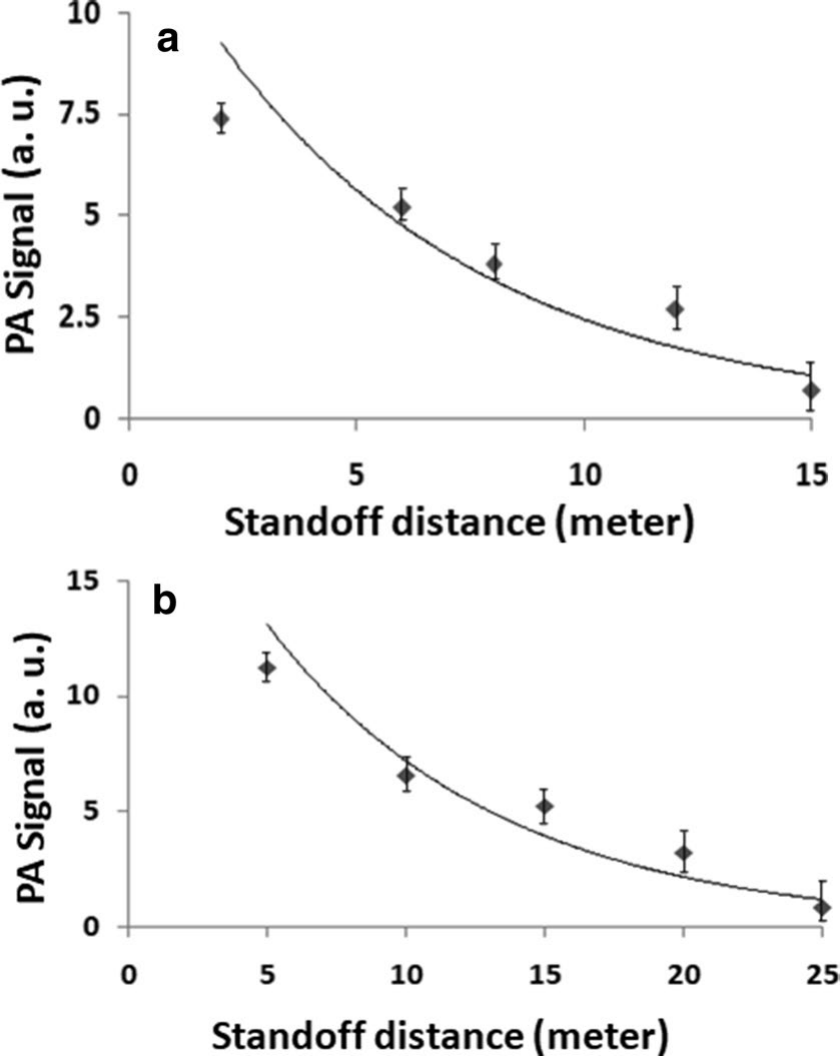
(**a**) Photoacoustic (PA) signals of TNT (5 μg/cm^2^), using only the QCL beam incident on the QTF, at varying standoff distances. (**b**) Photothermal (PT) signals of TNT (5 μg/cm^2^), using the QCL pump and 532 nm probe laser with QTF detector, at varying standoff distances.

The experimental setup similar to that of Fig. 2 was used to record the standoff photoacoustic (PA) spectra of TNT by using only the QCL source as described for the QE-LPAS technique^6^. In this case a gold coated mirror collecting mirror was used instead of enhanced aluminium coated mirror and a ZnSe focussing lens was used instead of glass lens. The variation of PA signal for TNT, with change in standoff distance is shown in Fig. 7a. It is found that the limit of PA signal detection is reached at 15 m for TNT residue concentration of 5 μg/cm^2^. In a parallel experiment of standoff photothermal (PT) detection; using a similar target as for the PA detection, the PT signal variation with distance is shown in Fig. 7b. It is found that the limit of detection for the PT signal is reached at ~ 25 m instead of 15 m (as in PA detection) for TNT concentration of 5 μg/cm^2^ on the target surface. A comparison of Fig. 7a and b shows that both the PA and the PT signals decay exponentially with increasing standoff distance. It is evident from these investigations that the PT detection, using the pump-probe laser technique, can be successfully employed at longer standoff distance in comparison to the QE-LPAS technique.

## Conclusion

Photothermal detection of hazardous chemicals has been demonstrated for the first time using the pump-probe laser beams coupled to the QTF detector. Standoff PT spectra have been recorded for TNT, RDX and Acetone. The PT-QTF sensor is capable of trace detection of chemicals adsorbed on surfaces of targets or in vapour/aerosol forms using tunable QCL in mid-IR region. The technology may be used to develop hand-portable product for forensic application and homeland security.
